# The Artery of Percheron Infarction after Coronary Angiography

**DOI:** 10.1155/2016/2402604

**Published:** 2016-04-26

**Authors:** Haitham Mazek, Khaled Sherif, Jose Suarez, Jason Wischmeyer

**Affiliations:** Internal Medicine Department, Texas Tech University Health Sciences Center, Lubbock, TX 79430, USA

## Abstract

Coronary angiography is the golden choice for coronary artery disease evaluation and management. However, as with any invasive procedures, there is a risk of complications. We are reporting a case of 69-year-old male with past medical history of cardiac bypass surgery, CHF, hypertension, and hyperlipidemia who was admitted to the hospital to evaluate his chest pain. He had treadmill stress test that showed ischemic induced exercise. Patient underwent coronary angiography that showed proximal complete occlusion of the RCA with a patent graft. At the end of the procedure, the patient did not wake up and remained minimally responsive. An urgent brain MRI was ordered and showed infarctions consistent with an artery of Percheron infarction. Later, patient has improved slowly and was discharged home. We briefly here discuss this rare complication including the risk factor, clinical presentation, and the management.

## 1. Introduction

Coronary angiography has been and currently remains the gold standard test for identifying the presence and extent of atherosclerotic coronary artery disease (CAD). This procedure is generally considered safe. However, as with any invasive procedure, there are specific patient-dependent and procedure-related complications associated with the test. One uncommon complication is stroke. We report a case of altered mental status following diagnostic coronary angiography.

## 2. Case

**Figure 1:**
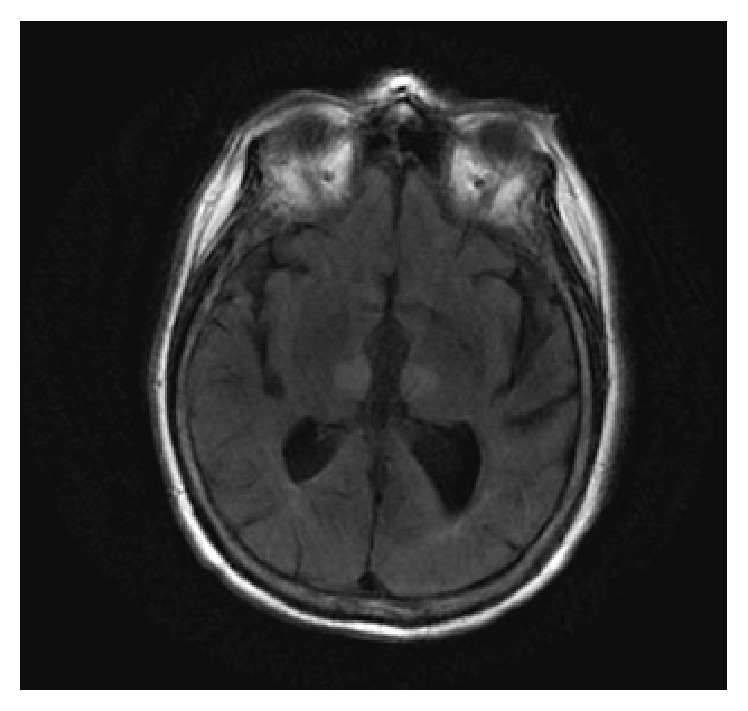
MRI with fast fluid-attenuated inversion showing hyperintensity at bilateral thalamic areas.

A 69-year-old man underwent coronary angiography for evaluation of chest pain. He had a treadmill stress test with myocardial perfusion imaging that revealed exercise-induced ischemia involving the inferior wall of the left ventricle. The patient has a history of cardiac bypass surgery, diastolic congestive heart failure, hypertension, and dyslipidemia. The angiography was done via right femoral artery using a 6-French arterial sheath. His coronary angiography showed proximal complete occlusion of the RCA with a patent graft. The procedure lasted 26 minutes during which the patient remained hemodynamically stable. During the procedure, he received 2 mg of midazolam and 1 mg of hydromorphone. At the end of the procedure, the patient did not wake up and remained minimally responsive. The patient withdrew from painful stimulation but had no spontaneous movements. One dose of naloxone 0.4 mg intravenous did not change his clinical examination. A neurology consultant reported that his pupils (left: 3 mm and right: 2 mm) were minimally reactive to light and that he had left medial rectus palsy. His reflexes were normal and there were no other focal deficits. His immediate CT scan of brain without contrast showed no acute hemorrhage or infarction. MRI of the brain confirmed bilateral medial thalamic and left midbrain infarctions consistent with an artery of Percheron infarction (Figure 1). The patient was continued on antiplatelet therapy. He did not receive TPA because of an arterial puncture at a noncompressible site within the last 7 days. The patient had slow improvement in his cognitive function. He became verbal and communicative but still not fully oriented. There was no improvement in the left third cranial nerve function; the limitation in vertical gaze movement was persistent. The patient was discharged after one week of hospitalization to a rehab center.

## 3. Discussion

Stroke after coronary angiography is uncommon but can be associated with substantial short- and long-term morbidity and mortality. It affects mainly elderly and high-risk patients with intraprocedural complications. Common symptoms include visual disturbance, motor weakness, aphasia, and altered mental status. Cerebral infarction following cardiac catheterization can be asymptomatic, and it occurs in about 15% of patients [1].

Predisposing factors for stroke include age, hypertension, diabetes mellitus, coronary angiography performed under emergency conditions, prior stroke, renal failure, the use of an intra-aortic balloon pump, and congestive heart failure [2]. Periprocedural strokes often occur during or immediately after the procedure when the femoral artery sheath is still in place, but the diagnosis can be delayed up to 36 hours in some cases [3]. Periprocedural stroke occurs in 0.03% to 0.3% of diagnostic procedures [3] and 0.3–0.4% of percutaneous coronary interventions (PCI) [2]. Cerebral microembolism is the main mechanism of periprocedural ischemic stroke occurring with PCI. Air embolism, thrombus formation in the catheter or on its surface, or dislocation of aortic atheroma during manipulation and passage of catheters within the aorta are the main sources of embolic material causing ischemic stroke during cardiac catheterization or PCI [4]. Al-Mubarak et al. showed that most of the embolic strokes during catheterization procedures are associated with an embolus located in either the common carotid bifurcation or the proximal middle cerebral artery [5]. In a recent 1-year study of pericoronary angiography strokes, the occluded vessel was the middle cerebral artery (MCA) in 24% of patients, the posterior cerebral artery in 19%, the basilar artery in 5%, the vertebral artery in 10%, and occlusion in 2 anterior circulation branches (MCA, anterior cerebral artery, or both) in 43% patients [6].

The artery of Percheron (AOP) is a small perforating artery or arteries supplying paramedian thalamus and midbrain. The incidence of AOP infarction is very low (from 0.1 to 2%) in all ischemic strokes due to variations of paramedian thalamic-mesencephalic arterial supply [7] and 4–18% in thalamic infarction [8]. The major mechanism of AOP infarction involves embolic or lacunar disease classified in many subgroups for prognosis [9]. Patients with bilateral paramedian thalamic strokes typically have a triad of altered mental status, vertical gaze palsy, and memory impairment like our patient's presentation. Altered mental status can present anywhere on the spectrum from drowsiness or confusion to hypersomnolence or coma. These disorders of vigilance generally occur with sudden onset and may persist until death. Some cases of complete recovery have been documented.

The management of stroke requires restoration of blood flow to the affected area of the brain as quickly as possible. In the case of intraprocedural stroke, the major advantages of performing a cerebral angiogram are that the diagnosis of embolic stroke can be confirmed and treatment can be given immediately. The occurrence of postprocedural stroke requires urgent cerebral imaging (well before 4.5 hours has elapsed) [10] to confirm an ischemic cause and to plan subsequent treatment. One of exclusion criteria for use of TPA is arterial puncture at a noncompressible site within 7 days [11].

Although stroke is uncommon, patients should be monitored closely throughout the procedure. In this case, endovascular therapy may have been the treatment of choice. However, recent studies did not show any benefit when compared to medical management [12]. It may be important for cardiovascular specialists to be familiar with cerebrovascular anatomy and angiographic technique to improve emergency care in patients with acute stroke.
